# Syphilis

**Published:** 1908-07

**Authors:** 


**Syphilis.** A Treatise for Practitioners. By **Edward L. Keyes**, Jr., A.B., M.D., Clinical Professor of Genitourinary Surgery in the New York Polyclinic Medical School and Hospital. Octavo, pp. 606. Illustrated. New York and London: D. Appleton and Company. 1908. (Cloth, $5.00.)

It is quite reasonable to assert that of all the books confined to syphilis this, of Keyes, is the most satisfying because it is most complete and because, further, it is based on clinical experience and case records extending over a period of forty years, in the practices of the author's father E. L. Keyes, Sr., and his fellow-worker Van Buren. This means that the results of over 2.500 cases have been dealt with. The disease is considered from every standpoint of its relation to public health, the various types of the disease being especially presented. What strikes one most forcibly in the book is the decisive manner of presentation of argument in favor of the author's treatment—though it is scarcely proper, perhaps, to designate the administration of protoiodide of mercury as the author's treatment, since it was first recommended and carried out by the elder Keyes some years ago, his first report of the then new treatment being followed by a whirl of protest and criticism which would have dismayed a less courageous man. The fact, however, that clinical experience has proven the wisdom and efficacy of his method, the tonic mercurial treatment, has proven the correctness of his views.

The author's style is clear; he does not believe this or that; nor does he think, perhaps or possibly; and the may-bes are cheerfully eliminated from his writing. What he says he says boldly and with conviction that he is right in his position as regards the management of syphilitic disease of whatever character, and the mass of cases reported from inception to discharge seems to bear out his attitude, which is one of favor toward the administration of the green protoiodide of mercury in preferance to the yellow.

One cannot overlook the completeness of the book. Every phase of syphilis is presented, its transmission, its immunity, course, prognosis of the various types and the special locations of attack, all receive most careful attention. There are, too, some firm statements which will come as a shock in greater or less degree to the general practitioner who treats syphilis as a routine procedure, on the cut and dried plan of first mercury to salivation and then iodide of potash in a sort of happy-go-lucky manner, until the patient either disappears or declares himself cured. One of these is that iodide of potash does not prevent relapses, but that mercury does; that for the early secondary symptoms mercury is the proper treatment and for all painful lesions of whatever period potash is indicated. The general everyday treatment of the disease will be improved if these vital points are remembered.

There is little in the book to criticise. The illustrations are excellent and the letter press is clean cut. In the chapter devoted to chancroid the author advises the use of carbolic acid as an anesthetic prior to cauterising the sore with nitric acid, and if the patient is nervous and the carbolic fails to produce the proper amount of insensibility then 10% cocaine solution may be used. It would appear that carbolic is unnecessary in any event. It is dangerous and there is no apparent contraindication to the use of cocaine in powder form for anesthesia of the surface to be cauterised. In cases of subpreputial chancroid and phimosis, Keyes recommends a dorsal or lateral incision and subsequent circumcision after the cure of the ulcers. A more rapid method, and one which is wholly satisfactory, is the circumcision with the actual cautery under general anesthesia; this not only destroys the ulcers completely but makes a secondary operation for removal of the mutilated prepuce unnecessary.

In the treatment of bubo he recommends several small incisions and the injection into the mass of iodoform in glycerine; then, "if after several weeks the indurated mass remains it must be excised." Excision, he warns, leaves a wound which may and usually does take, one infers, several months to heal. This is not borne out by clinical experience in the writer's services. At any rate, better results will be secured if when the bubo is opened and thoroughly cleansed it be packed with 3% collargolum gauze.

The book deserves the position which it has taken in medical literature,—that of the best exposition of syphilis which has yet been published.

